# Polypharmacology of clinical sodium glucose co‐transport protein 2 inhibitors and relationship to suspected adverse drug reactions

**DOI:** 10.1002/prp2.867

**Published:** 2021-09-29

**Authors:** Karan Matharu, Kiran Chana, Charles J. Ferro, Alan M. Jones

**Affiliations:** ^1^ School of Pharmacy Institute of Clinical Sciences College of Medical and Dental Sciences University of Birmingham Birmingham United Kingdom; ^2^ Birmingham Cardio‐Renal Group Institute of Cardiovascular Sciences College of Medical and Dental Sciences University of Birmingham Birmingham United Kingdom

**Keywords:** adverse drug reactions, polypharmacology, SGLT2

## Abstract

Sodium glucose co‐transporter 2 inhibitors (SGLT2i) are a promising second‐line treatment strategy for type 2 diabetes mellitus (T2DM) with a developing landscape of both beneficial cardio‐ and nephroprotective properties and emerging adverse drug reactions (ADRs) including diabetic ketoacidosis (DKA), genetic mycotic infections, and amputations among others. A national register study (MHRA Yellow Card, UK) was used to quantify the SGLT2i's suspected ADRs relative to their *Rx* rate (OpenPrescribing, UK). The polypharmacology profiles of SGLT2i were data‐mined (ChEMBL) for the first time. The ADR reports (*n* = 3629) and prescribing numbers (*R_x_ n* = 5,813,325) for each SGLT2i in the United Kingdom (from launch date to the beginning December 2019) were determined. Empagliflozin possesses the most selective SGLT2/SGLT1 inhibition profile at ~2500‐fold, ~10‐fold more selective than cangliflozin (~260‐fold). Canagliflozin was found to also inhibit CYP at clinically achievable concentrations. We find that for overall ADR rates, empagliflozin versus dapagliflozin and empagliflozin versus canagliflozin are statistically significant (*χ* ^2^, *p* < .05), while dapagliflozin versus canagliflozin is not. In terms of overall ADRs, there is a greater relative rate for canagliflozin > dapagliflozin > empagliflozin. For fatalities, there is a greater relative rate for dapagliflozin > canagliflozin > empagliflozin. An organ classification that resulted in a statistically significant difference between SGLT2i was suspected infection/infestation ADRs between empagliflozin and dapagliflozin. Our findings at this stage of SGLT2i usage in the United Kingdom suggest that empagliflozin, the most selective SGLT2i, had the lowest suspected ADR incident rate (relative to prescribing) and in all reported classes of ADRs identified including infections, amputations, and DKA.

## Graphical abstract

At this stage of prescribing in the UK, the lowest percentage of suspected adverse drug reactions (ADRs) and fatalities was found with Empagliflozin

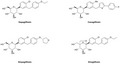

**Abbreviations**
ADRs: adverse drug reactions
AKI: acute kidney injury
CKD: chronic kidney disease
DKA: diabetic ketoacidosis
SGLT2i: Sodium glucose co‐transporter 2 inhibitors
T2DM: type 2 diabetes mellitus

## 1 INTRODUCTION

Sodium glucose co‐transporter 2 inhibitors (SGLT2i) are a relatively new class of antidiabetic medication for type 2 diabetes.^1^, ^2^ At the time of writing, four SGLT2i have been approved for use in clinical practice in the United Kingdom (approval date in parentheses): dapagliflozin (2012), canagliflozin (2013), empagliflozin (2014), and ertugliflozin (2019), respectively.

In comparison to other antidiabetic medication,^3^ the pharmacological action of SGLT2i works independently of insulin.^4^, ^5^, ^6^ SGLT2i competitively bind to SGLT2, are predominantly found in the kidneys, and are responsible for 90% of glucose reabsorption in the body, leading to the inhibition of this transporter. This consequently results in glucosuria and beneficial changes can be observed metabolically and hemodynamically in the body.^5^, ^7^, ^8^

The inhibition of SGLT2 leads to the reduction in blood pressure, body weight,^9^ and increase in ketone production. The increase in plasma ketones is associated with increased glucagon secretion from the pancreas, as a response to reduced glucose and lipolysis. The heart utilizes the ketones for fuel and produces more ATP per molecule compared to glucose, decreasing oxidative stress. Diabetes is associated with both macrovascular and microvascular complications, an example being diabetic nephropathy which is caused by damage to blood vessels due to high blood glucose. Another indirect action of SGLT2 inhibition is natriuresis; this further reduces blood pressure and slows down the progression of chronic kidney disease (CKD).^6^ Thus, SGLT2i have both cardioprotective and nephroprotective features.^7^, ^8^, ^10^ This has been supported by the following EMPA‐REG OUTCOME (Empagliflozin Cardiovascular Outcome Event Trial in Type 2 Diabetes Mellitus Patients), DAPA‐HF (Dapaglifozin and Prevention of Adverse Outcomes in Heart Failure), and CANVAS (the CANagliflozin cardioVascular Assessment Study) trials.^5^, ^6^, ^11^, ^12^, ^13^, ^14^, ^15^ Collectively, the results of the landmark trials demonstrated that SGLT2i correspond to a reduction in all cardiovascular mortality risks, reduced heart failure‐associated hospitalization, and decline in CKD progression in diabetic patients.^16^ These properties were identified to be shared across the SGLT2i class and therefore highlight the potential use in other co‐morbidities including non‐diabetic CKD.^17^

Adverse drug reactions (ADRs) are defined as harmful and unintended events in response to a drug related to any dose.^18^ SGLT2i are associated with ADRs^19^ such as diabetic ketoacidosis (DKA),^20^ acute kidney injury (AKI), genital mycotic infections, limb amputations and emerging links to stroke,^21^ and bladder cancer.^22^, ^23^ However, there is no research regarding the polypharmacology of SGLT2i^24^, ^25^ and their suspected ADRs in pharmacovigilance studies.^26^, ^27^ This research aims to identify potential links between ADRs for the three most established SGLT2i in the United Kingdom with their unique polypharmacology and physicochemical profiles.

Our continuing research interest in the intersection of medicinal chemistry^28^ with clinical prescribing and associated ADRs^29^, ^30^, ^31^ warranted an application of our techniques for understanding the potential links between the SGLT2i's polypharmacology and ADRs landscape. Furthermore, recent studies have revealed that drugs believed to be highly selective frequently address multiple target proteins^32^ and this may affect SGLT2i performance^33^ and could lead to a more stratified approach in clinical practice.

## 2 MATERIALS AND METHODS

### 2.1 Chemical and pharmacokinetic properties of SGLT2 inhibitors

Physicochemical properties are intrinsic to each SGLT2i and have an impact on how they behave in the body including permeability, clearance, and absorption.

The Electronic Medicines Compendium^34^ and ChEMBL database^35^, ^36^, ^37^, ^38^ were used to identify the chemical properties and pharmacokinetics of the SGLT2i (Table 1). Physicochemical properties including molecular weight, *p*Ka, Log_10_P, and Log_10_D values were datamined from ChEMBL using the search terms of each drug's name. Calculated properties were obtained using the ChemDraw 19.0 software based on the chemical structure of each drug.

**TABLE 1:** The physiochemical and pharmacokinetic properties for the four SGLT2i

Properties	Empagliflozin	Dapagliflozin	Canagliflozin	Ertugliflozin
Dose (mg)	10	10	100	5
Bioavailability (%)	≅78	≅78	≅65	≅100
*C*_max_ (nM)	259.0	387.1	2767.1	186.4
Route	Oral	Oral	Oral	Oral
Dosage form	Tablet	Tablet	Tablet	Tablet
Half‐life (h)	≅13.1	≅12.9	10.22–13.26	11–18
Clearance (mL/min)	176.6	207	192	185–215
Volume of distribution (L)	73.8	118	83.5	85.5
PPB (%)	86.2	91	99	94–95
MW (Da)	450.14	408.87	444.5	436.8
*p*Ka	12.57	12.57	13.34	11.98
HB acceptors	7	6	5	7
HB donors	4	4	4	4
*^t^* PSA (Å)	108.61	99.38	90.15	108.61
Log_10_P	2.51	2.11	3.44	3.19
Log_10_D	2.51	2.11	3.44	3.19
IC_50_ (nM)	3.1	1.2	2.7	0.9
*p*IC_50_	8.51	8.92	8.57	9.06
LLE	6.00	6.22	5.13	5.87

Clearance, a measure of how rapidly a drug is excreted; HB, hydrogen bond(s); *^t^* PSA, topological polar surface area of the compound.

Neutral or basic–acidic drug molecules are defined by the *p*K_a_ (negative base10 logarithm of the acid dissociation constant; the lower the *p*K_a_ value the stronger the acid); log_10_P (where *P* is the partition coefficient = concentration of solute in octanol divided by the concentration of solute in water); log_10_D is the distribution constant of a drug between the aqueous and lipid phases at *p*H 7.4.

The following parameters were calculated; *p*IC_50_ was calculated using the median SGLT2 inhibitory IC_50_ of each drug; and lipophilic ligand efficiency (LLE) was calculated: LLE = *p*IC_50_–clog_10_P. Lipophilic ligand efficiency (LLE) measures how effective a drug binds to its target excluding nonspecific entropic factors. The LLE parameter is used to normalize potency relative to lipophilicity. A value of <5 is associated with increased toxicity.^39^, ^40^, ^41^ The threshold for BBB penetration was set as: molecular weight <450 Da; <6 hydrogen bond donors (HBD); <2 hydrogen bond acceptors (HBA); neutral or basic drug molecule (defined by *p*Ka); topological polar surface area (*^t^* PSA) <90 Å; logD_7.4_ 1–3; and low affinity to efflux ABCB1 (P‐glycoprotein, MDR1). The *C*_max_ peak serum concentration of each SGLT2i was calculated from the FDA new drug application documentation.^42^, ^43^, ^44^, ^45^

Non‐listed pharmacokinetic parameters on the ChEMBL database were collated through literature database searches of each drug name + pharmacokinetic OR PK search terms using Springer Link; NCBI; the electronic Medicines Compendium (eMC); and SciFinder (Caplus and Medline databases).^46^, ^47^, ^48^, ^49^, ^50^, ^51^, ^52^

### 2.2 Pharmacological properties of SGLT2 inhibitors

Pharmacological bioactivity data were curated. The ChEMBL database (accessed on 01/11/2020) was used to gather quantitative measures between each SGLT2i and human proteins. SGLT2 activity data were also identified through cross‐checking literature using the Reaxys^®^ database. Bioactivity was compared using IC_50_ values, with a minimum threshold set at 10 μM (to exclude weak interactions). IC_50_ is a quantitative measure that indicates how much of a substance (in this case a drug) is required to inhibit a biological component by 50%.

The mean IC_50_ gives an overview of the relative affinity between SGLT2i across multiple targets and mitigates for reproducibility/reliability issue of selecting a single IC_50_.

Additional polypharmacological inhibitory data were collected through Food Drug Administration (FDA) Secondary Pharmacology Data Review Documents.^42^, ^43^, ^44^, ^45^, ^53^

### 2.3 Nomenclature of targets and ligands

Key protein targets and ligands in this article are hyperlinked to corresponding entries in http://www.guidetopharmacology.org and are permanently archived in the Concise Guide to PHARMACOLOGY 2019/20.^54^, ^55^

### 2.4 Yellow card scheme

Reported suspected ADR data were extracted from the Medicines and Healthcare Regulatory Agency (MHRA) Yellow Card Interactive Drug Analysis Profile in the United Kingdom.^23^ Adverse patient event data were curated from the Yellow Card Scheme Interactive Drug Analysis Profiles web portal (https://yellowcard.mhra.gov.uk/iDAP/), from January 2013 to December 2019 for the studied SGLT2i. The ADR event data collection was terminated in January 2020 due to differences in reviewal dates between the Yellow Card Report Scheme and Open Prescribing.

The reporting system processed reports for all SGLT2i except for ertugliflozin. Ertugliflozin has only recently been approved for use in clinical practice (2019), and thus, no ADR data was available at the time of data collection. The Yellow Card Report scheme categorized ADRs by organ class, which was replicated in this study.

Significant ADRs were selected and assessed within this study. The selection criteria included differential ADRs across the SGLT2i (independent of ADR level above baseline) or high levels of ADR within a particular organ class (above baseline). The ADR table included ADRs that are established and suspected ADRs that were reported (*n* > 50) for each SGLT2i (Chart S1). ADRs reported under the investigations system organ class, non‐serious ADRs, or ADRs because of multiple active constituents were removed to avoid biases.

The ADR data required standardization to allow for comparisons between the different drugs. ADRs per 100,000 *R_x_* is a standard approach in signal hypothesis generation.^29^, ^31^

### 2.5 Prescribing data

The Open Prescribing (https://openprescribing.net/) database collects data regarding prescription numbers for all prescribed drugs in England across all National Health Services (NHS) in primary care settings.^56^ The data for prescription numbers were extracted between 2013 and 2019 for canagliflozin and 2014–2019 for dapagliflozin and empagliflozin, respectively. The difference in start date coincides with the first prescribing date recorded for each SGLT2.

### 2.6 Statistical analysis

Chi‐squared (*χ* ^2^) test was performed on the standardized ADR/100,000*R_x_* data to determine statistically significant differences between the suspected ADRs and SGLT2i using Excel for Microsoft 365. A *p*‐value of <.05 was considered statistically significant.^57^ Ertugliflozin was excluded from this part of the study due to the small number of reports.^58^ Statistical analysis was performed on the three established SGLT2i (empagliflozin, dapagliflozin, and canagliflozin). As we are interested in (1) differences between the SGLT2i and not as to whether a particular ADR is related to SGLT2i therapy and (2) because of the exploratory nature of this study, the lack of data on potential confounders, and the relatively low incidence of some of the ADRs, we used raw data rather than disproportionate analysis. The exploratory nature of this study also meant that corrections for multiple comparisons were not used.

### 2.7 Ethic approvals and consent to participate

This study was conducted using publicly available data and no patient identifiable data was used. Thus, there was no requirement for ethical approval and approval of consent was required.

## 3 RESULTS

### 3.1 Chemical properties and pharmacokinetics

**FIGURE 1:**
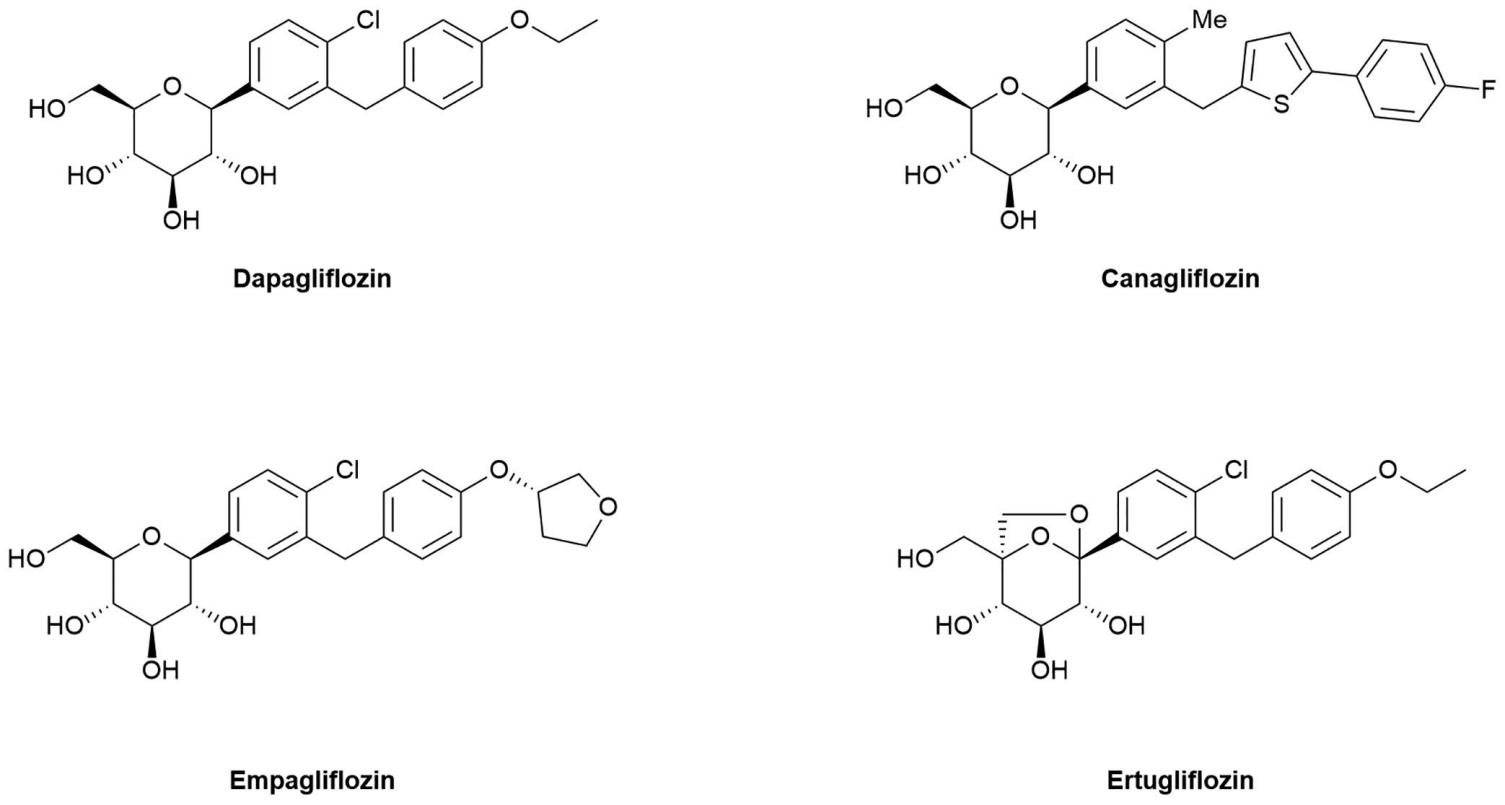
Molecular structures of the SGLT2i used in this study

The physical properties of all SGLT2i are similar, all being *C*‐glycosides (Figure 1). They have prolonged action in the body due to the long *t*_½_ and decreased metabolism in the gastrointestinal (GI) system. All SGLT2i are given orally and as single‐dose medication (Table 1). Volume of distribution (*V*_d_) is similar across all SGLT2i (73.8–85.5 L), the exception being dapagliflozin which has *V*_d_ of 118 L indicating its presence in tissues is higher than other class members.

### 3.2 Pharmacological properties

IC_50_ > 10 µM is considered a weak inhibitor and thus is clinically unachievable in most scenarios. SGLT2i *C*_max_ range from 186 to 387 nM except for canagliflozin (2.8 µM).

Exhaustive data regarding the polypharmacology of the SGLT2i are available in Table S2 and key data are presented in Table 2.

**TABLE 2:** The pharmacology data of the four available SGLT2i

Inhibitory activities/drug	Empagliflozin	Dapagliflozin	Canagliflozin	Ertugliflozin
SGLT (SLC5A gene)				
SGLT2 IC_50_ (nM)	3.1	1.2	2.7	0.87
Selectivity (SGLT2:SGLT1)	≅2500 fold	≅1200 fold	≅260 fold	≅2200 fold
SGLT1 IC_50_ (nM)	8300	1400	710	1960
SGLT4 IC_50_ (nM)	11,000	9100	7900	‐
SGLT5 IC_50_ (nM)	1100	820	1700	‐
SGLT6 IC_50_ (nM)	2000	1300	240	‐
CYP450 isoforms				
CYP2D6 IC_50_ (nM)	>150,000	>40,000	1320	‐
Organic anion transporters				
OATP1B3 IC_50_ (nM)	58,600	8000	‐	1,50,700

(‐) represents no inhibitory data was reported.

The primary pharmacological action of SGLT2i is the inhibition of SGLT2. Comparing SGLT2 inhibitory values, ertugliflozin is the most potent inhibitor (IC_50_ = 0.87 nM) and empagliflozin the weakest (IC_50_ = 3.1 nM).

The pharmacological activity includes inhibition of other SGLT isoforms (1, 4, 5, and 6). Inhibition of these transporters are weaker in comparison to the on‐target (>1000 nM) except for dapagliflozin's inhibition of SGLT5 (IC_50_ = 820 nM) and canagliflozin's inhibition of SGLT6 (IC_50_ = 240 nM). SGLT2i are weak inhibitors of CYP_450_ isoforms, with exception of canagliflozin which inhibits CYP2D6 (IC_50_ = 1320 nM).

Selectivity values were approximations that compared inhibitory activity at SGLT2 versus SGLT1. Differences in selectivity between SGLT2is were noted.^59^, ^60^ Canagliflozin, selectivity being around 260‐fold and can be considered the least selective and empagliflozin, being the most selective ~2500‐fold for SGLT2 (Table 2).

Based on the physicochemical and polypharmacological data available, the following predictions can be made: empagliflozin is likely to emerge as the SGLT2i with the least number of serious ADRs due to high selectivity for SGLT2.

### 3.3 Adverse drug reactions (ADRs)

Prescription rates between SGLT2i differed; empagliflozin had the highest (2,818,343) and canagliflozin had the lowest (899,872). To enable a fair comparison between SGLT2i with a wide range of prescribing numbers, standardized values were calculated (ADR per 100,000 *R_x_*) as shown in parentheses (Table 3). The standardization of suspected ADR data to prescribing rate prevents misinterpretation of the raw values and has been applied in other pharmacovigilance studies.^29^, ^31^

**TABLE 3:** Summary of the adverse drug reaction for three established SGLT2i in the United Kingdom

	Empagliflozin	Dapagliflozin	Canagliflozin	*p* value
Total number of *R_x_*	2,818,343	2,095,110	899,872	
Total number of ADRs	1242 (44.07)	1651 (78)	736 (82)	<.005
Total number of fatalities	6 (0.213)	19 (0.907)	5 (0.562)	.807
Gastrointestinal system				
Total ADRs	172 (6.10)	232 (11.07)	111 (12.34)	.331
Nausea and Vomiting	73 (2.59)	83 (3.96)	43 (4.78)	.723
General system disorders				
Total ADRs	134 (4.75)	238 (11.36)	103 (11.45)	.200
Asthenic conditions	60 (2.13)	88 (4.20)	34 (3.78)	.701
Hepatobiliary disorders				
Total ADRs	8 (0.284)	20 (0.955)	5 (0.556)	.827
Infections and infestations				
Total ADRs	130 (4.61)	206 (9.83)	139 (15.45)	.052
Fungal infections	33 (1.17)	62 (2.96)	30 (3.33)	.584
Urinary tract infections	21 (0.75)	50 (2.39)	51 (5.67)	.118
Fournier's gangrene	10 (0.35)	14 (0.67)	4 (0.44)	.274
Renal and urinary disorders				
Total ADRs	105 (3.73)	199 (9.50)	75 (8.33)	.274
Urinary disorders	70 (2.48)	123 (5.87)	52 (5.78)	.452
AKI	22 (0.78)	32 (1.53)	15 (1.67)	.841
Reproductive system				
Total ADRs	41 (1.45)	75 (3.58)	18 (2.00)	.593
Penile disorders	13 (0.46)	8 (0.38)	3 (0.33)	.989
Balanoposthitis	8 (0.28)	9 (0.43)	3 (0.33)	.983
Vulvovaginal disorders	8 (0.28)	22 (1.05)	7 (0.78)	.805
Surgical and medical procedures				
Total ADRs	15 (0.53)	12 (0.57)	17 (1.89)	.548
Foot amputations	0	0	1 (0.11)	.368
Limb amputations	2 (0.07)	1 (0.05)	0	.368
Toe amputation	7 (0.25)	5 (0.24)	13 (1.44)	.477
Leg amputation	1 (0.035)	3 (0.14)	0	.913
Nervous system				
Total ADRs	96 (3.41)	159 (7.59)	70 (7.78)	.377
Neurological disorders	66 (2.34)	106 (5.06)	48 (5.33)	.525
Metabolic disorder				
Total ADRs	381 (13.52)	535 (25.54)	208 (23.11)	.307
DKA	271 (9.62)	337 (16.09)	117 (13.00)	.444
EuDKA	36 (1.28)	27 (1.29)	24 (2.67)	.693
Musculoskeletal disorders				
Total ADRs	46 (1.63)	93 (4.44)	47 (5.22)	.388
Back pain	11 (0.39)	23 (1.10)	3 (0.33)	.739

Numbers in parentheses are ADRs per 100,000 *R_x_*. The *p* value was obtained using *χ^2^* analysis.

Abbreviations: AKI, acute kidney injury; DKA, diabetic ketoacidosis; EuDKA, euglycemic diabetic ketoacidosis.

### 3.4 All ADRs

Canagliflozin had the highest ADR rate per 100,000 *R_x_* (82), followed by dapagliflozin (78) then finally empagliflozin (44.07); this discrepancy resulted in a *p* < .05. A *p* < .05 was present for both the comparison between empagliflozin and dapagliflozin and the comparison between empagliflozin and canagliflozin but not dapagliflozin versus canagliflozin (Table S1).

The calculated *p* values for ADRs per individual organ class for all SGLT2i were >.05 for all ADRs by organ class/100,000 *R_x_* (Table 3). Overall, no statistical differences emerged in the respective organ classes. However, dapagliflozin and canagliflozin had similar ADR trends to each other, with empagliflozin being lower in number of ADRs across the table but not sufficiently so at the time of this research, to warrant a statistically significant conclusion.

### 3.5 ADR summary

Dapagliflozin had the highest fatality rate per 100,000 *R_x_* (0.907), followed by canagliflozin (0.562) and empagliflozin (0.213); further details are shown in Chart S2.

The ADRs reported for GI system disorders were relatively high for all SGLT2i. Canagliflozin had the highest incident rate of 12.34, approximately two‐fold greater than empagliflozin (6.10). Dapagliflozin (11.07) had a similar ADR incident rate to canagliflozin, showing a differential between empagliflozin and the other SGLT2i.

The incident rate for Fournier's gangrene, an ADR that has been reported to have an increased association with SGLT2i,^61^, ^62^, ^63^ was similar between all SGLT2i studied (0.35–0.67). Dapagliflozin had the highest ADRs of the renal and urinary system (9.50), followed by canagliflozin (8.33) and empagliflozin the lowest (3.73). However, for urinary disorders, both canagliflozin and dapagliflozin had a similar incidence rate, 5.78 and 5.87, respectively, whereas the empagliflozin incident rate was 2.48.

Canagliflozin had the highest rate for amputations (1.17) when compared to empagliflozin and dapagliflozin where the incidence was observed to be similar, 0.53 and 0.57, respectively. Canagliflozin use was associated with toe (1.44) and foot amputations (0.11). However, empagliflozin and dapagliflozin use was associated with foot, toe, and leg amputations at a relatively low incidence. The ADR incident range for toe and leg amputations for both empagliflozin and dapagliflozin was 0.24–0.25 and 0.035–0.14, respectively.

For all ADRs relative to the nervous system, dapagliflozin (7.59) and canagliflozin (7.78) had a two‐fold higher incidence of ADRs when compared to empagliflozin (3.41). This trend was repeated for neurological disorders, where empagliflozin had the lowest incidence (2.34) and both dapagliflozin and canagliflozin incidence was relatively similar (>5).

For all ADRs relative metabolic disorders, empagliflozin had the lowest incidence (13.52) which was substantially different to both dapagliflozin and canagliflozin, 25.54 and 23.11, respectively. However, for DKA, the differential range between all three SGLT2i was considerably smaller as this ADR is prevalent with all of them (9.62–16.09). This corresponded to empagliflozin having the lowest and dapagliflozin having the highest. For euglycemic diabetic ketoacids, incidence was similar in both dapagliflozin and empagliflozin; however, in canagliflozin, the incidence was greater (2.67).

## 4 DISCUSSION

The aim of this research was to identify potential relationships between the pharmacological activity of SGLT2i with their suspected ADRs. Visual inspection of Table 3 reveals a recurring trend in which the ADR incident rate increased across the table (from empagliflozin to canagliflozin). This corresponded to a decrease in selectivity as shown in Table 2 suggesting a potential relationship between the selective nature of SGLT2i and their respective ADR signal profile. It should also be noted that not all pharmacological targets’ inhibition is clinically achievable (based on each drug's respective *C*_max_), indeed only canagliflozin is likely to be able to inhibit off‐targets SGLT1, SGLT6, and CYP isoforms potently in man.

### 4.1 SGLT2 inhibition in relation to ADRs

The polypharmacology of SGLT2i was predominantly of other SGLT transporters. In total, there are six SGLT transporters all coded by the SLC5A gene, but their functions and whereabouts in the body differ.^64^ SGLT1 is located in the kidneys and is also present in the heart, small intestine, brain, and liver. In the kidneys, SGLT1 is responsible for 10% of glucose reabsorption from the urine. SGLT1 inhibition in the intestine is associated with GI side effects.^65^, ^66^ However, the roles of SGLT1 in other organs are not well understood.

The roles of the remaining SGLT transporters (SGLT4‐6) are not fully elucidated to the best of our knowledge except for SGLT3 which is not a monosaccharide transporter but instead a sensor in the brain.^67^, ^68^ Therefore, no potential relationships can be drawn between the inhibition of SGLT1 at different organ levels (except the kidneys) and other SGLT transporters with their suspected adverse effects.

A recent study proposed that as a compensatory effect of direct SGLT2 inhibition, SGLT1 is upregulated in the kidneys and becomes responsible for approximately 40% of glucose reabsorption rather than the accepted 10%.^65^, ^66^ Due to the pharmacological activity of SGLT2i on SGLT1, SGLT1 inhibition may eventually lead to an increased glucosuria effect that could potentially be related to numerous complications such as ketoacidosis, limb amputations, and genital mycotic infections. The intensity of glucosuria is therefore related to the selectivity profile for SGLT2i. Canagliflozin's low selectivity due to its strong inhibitory effect on SGLT1 may correspond to an intense glucosuria effect. Whereas empagliflozin's high selectivity for SGLT2 may correspond to the lower comparative intensity in glucosuria, which is supported by the lower ADR incident rate for all ADRs, 44.07 and *p* < .05 (Table 3).

Complications that may arise due to increased urinary glucose excretion due to inhibition of both SGLT2 and SGLT1 eventually lead to indirect changes in the body. The indirect action of SGLT2 inhibition in the kidneys is associated with natriuresis.^4^ As SGLT1’s pharmacological action in the kidneys is analogous to SGLT2, inhibition of both could lead to potential increase in natriuresis. As a result of osmotic diuresis, loss of plasma volume and thus a reduction in blood pressure occurs. Therefore, this could explain the increased lower limb amputation rate associated with SGLT2i that is the result of hypovolemia and decreased tissue perfusion. Despite the association with all SGLT2i, canagliflozin had a greater relative amputation rate (compared to other SGLT2i) potentially due to its SGLT1 potent inhibition.^69^, ^70^, ^71^

Furthermore, higher SGLT1 inhibitory activity with an SGLT2i corresponds to a higher infection rate which can be observed in Table 3 that displays canagliflozin possess the highest incident rate. The potential mechanism that leads to a greater incidence in infection, both urinary tract and fungal infections, could be related to increase glucose presence in the urine. The environment is more nutrient rich and preferable for bacteria growth in the urinary tract and for fungus. Coupled with other urinary disorders such as urine dysfunction, polyuria, nocturia, incomplete bladder emptying, and weakened immune system due to diabetes may explain the high incidence of infection.^72^ The concomitant usage of dipeptidyl peptidase‐4 inhibitors is known to moderate the risk of genitourinary tract infections with SGLT2i use.^73^

An increase in glucose excretion in urine is indirectly related to an increase in glucagon secretion from alpha cells in the pancreas. This response is due to negative feedback in glucose control, reduced plasma glucose leads to glucagon secretion, and is an indirect action of SGLT2i. Glucagon is responsible for gluconeogenesis and glycolysis to regulate plasma glucose. The by‐product of this is an increase in ketones in the plasma. Elevated ketones in the plasma elicit oxidative stress; other inflammatory responses thus can be potentially fatal. Other concerns include metabolic acidosis, as ketones remove bicarbonates in the body which is potentially hazardous.^74^, ^75^

The Yellow Card pharmacovigilance scheme has already provided an important safety update regarding SGLT2i and ketone levels.^76^ SGLT2 inhibitor treatment should be interrupted in patients who are hospitalized for major surgical procedures or acute serious medical illnesses and ketone levels measured, preferably in blood rather than urine. Treatment may be restarted when the ketone values are normal and the patient's condition has stabilized.

Euglycemic ketoacidosis is a type of DKA and is a mixture of metabolic acidosis and hyperglycemia.^77^ The relevance of increased ketone production in relation to SGLT2i despite it being prevalent for all is that dapagliflozin directly increases glucagon secretion in hyperglycemic conditions. This was observed in a study that compared glucagon secretion in mouse cells using canagliflozin and dapagliflozin. The pancreatic cells in mice are like humans. In both human and mice cells, SGLT1 is expressed on the alpha cells and inhibition of which suppresses glucagon secretion and was observed with canagliflozin due to potent inhibitory effect of SGLT1.^78^, ^79^ Dapagliflozin directly causing glucagon secretion from the pancreas may explain why the incident rate for DKA and total metabolic disorders were the highest, 16.09 and 25.54, respectively (Table 3). All SGLT2i indirectly cause glucagon secretion, due to urinary glucose excretion.

### 4.2 Other transporter inhibition in relation to ADRs

All SGLT2i are weak inhibitors of other biological targets. Only canagliflozin produced a strong enough inhibitory effect on CYP450 isozyme—2PD6; however, they play a minimal role in the metabolism and excretion of this SGLT2i.^4^, ^80^ All SGLT2i are predominantly excreted in urine or metabolized by uridine 5'‐diphospho‐glucuronosyltransferase to form metabolites which are then excreted in the urine.^81^ CYP2D6 is expressed in the liver and some parts of the brain but due to CYP enzymes expressing an insignificant role in elimination of SGLT2i, it is unclear the link, if any, to the ADRs. There is not a significant difference between the half‐life of canagliflozin and other SGLT2i (Table 1). Normally, the inhibition of CYP enzymes eventually leads to an increase in half‐life, so the drug will stay present in the body longer and produces an extended pharmacological action. Inhibition would also precipitate drug to drug interactions as CYP enzymes are responsible for the elimination of most drugs.

In addition, dapagliflozin inhibited organic anionic transporter polymorph (OATP1B3) which is responsible for numerous drugs transportation in the body and consequences of inhibition may lead to increase drug to drug interactions.^82^ This poses as a possible risk in prescribing in clinical practice, as complications of drug–drug interactions can vary.

Furthermore, OATP1B3 is a transporter for bilirubin and inhibition of which is related to hyperbilirubinemia can lead to jaundice, cholestasis, and abnormal liver function. Hyperbilirubinemia is associated with neurological damage and can be fatal. This may explain why incidence of neurological disorders and hepatobiliary disorders are more prevalent with dapagliflozin than other SGLT2i (Table 3). Furthermore, as glucose is the main metabolite for the brain for energy, varying plasma glucose may have negative implications on the nervous system.

### 4.3 Limitations

Reported SGLT2i ADRs were sourced through the MHRA Yellow Card reporting scheme. The MHRA Interactive Drug Analysis Profiles (iDAP) give a complete listing of all the spontaneous suspected ADRs reported through the Yellow Card Scheme. While a valuable safety tool, there are several inherent limitations: underreporting of suspected ADRs is commonly encountered and may lead to the underestimation of any given reaction or drug even within the same class.^83^ Furthermore, publicity about an adverse effect,^84^ length of time on the market, and novelty of the drug may also affect reporting. This can mean that comparisons between drugs using such reports can be problematic, particularly when small numbers are involved. Declines in reporting ADRs after the second year of a drug on the market, known as the Weber effect, have been reported.^85^

Reporters are requested to report any suspected ADRs, and they do not have to demonstrate a clear causal link with the drug. These reported adverse reactions have not been proven to be related to the SGLT2i and should not be interpreted as a list of known side effects. Care needs to be taken with suspected fatal cases, where reporters may be more likely to err on the side of reporting due to the seriousness of the reaction. Other factors would need to be considered: co‐morbidities, co‐medications, genetics, and others. Therefore, some of these suspected ADRs may be due to third factor variables. Therefore, conclusions on the safety and risks of medicines cannot be made on the information obtained from the Drug Analysis Profiles alone. However, such data can be useful for hypothesis generation as in this study, standardized to prescribing rates, and supported by primary literature reports and case studies.

The available data on the polypharmacology of SGLT2i is incomplete.^86^, ^87^ For instance, there is also a lack of information regarding the inhibition of SGLT1 at various organ levels (except the kidneys and intestine) and inhibition of other SGLT transporters which may have unintended consequences. In this work, we have mitigated this risk by performing data integration of the pharmacological effects of SGLT2i integrating public databases, literature, and NDA documentation. However, not all SGLT2i had been comprehensively tested against all the targets we identified and their interactions with other target families remain unknown.

## 5 CONCLUSION

The results demonstrate a correlation between the selectivity of SGLT2i in relation to their respective suspected incidence of ADRs. As a class, SGLT2i are a safe and effective medication for the treatment of T2DM with relatively low incidence of ADRs and fatalities and may have potential for the treatment for other diseases (cardio‐ and nephroprotective properties).

Empagliflozin emerged as the most prescribed SGLT2i in the United Kingdom, the most selective SGLT2i on the market, and has the lowest incidence rate of suspected ADRs across the SGLT2i class. For established ADRs, empagliflozin had both a relatively low incidence for all ADRs (44.07), infections (4.61), amputations (0.53), and diabetic ketoacidosis (9.62) per 100,000 *R_x_*. Although a statistically significant difference between the SGLT2i could not be determined, empagliflozin consistently had the lowest ADR incidence out of all the studied SGLT2i.

Due to the possible linkage between SGLT2i selectivity and incidence of suspected spontaneous ADRs, we would predict that ertugliflozin will have a similar ADR profile as empagliflozin (as data emerge), because of the drug's high selectivity (≅2200‐fold for SGLT2 over SGLT1).

## DISCLOSURE

No conflict of interest to report.

## Supporting information

Supplementary Material

## Data Availability

The data that support the findings of this study are available in the supplementary material of this article.

## References

[1] Joint Formulary Committee . British National Formulary [Internet]. British Medical Association and Royal Pharmaceutical Society of Great Britain. Joint Formulary Committee. British National Formulary [online]. London: BMJ Group and Pharmaceutical Press. Accessed November 12, 2020. http://www.medicinescomplete.com

[2] Bhattacharya S , Rathore A , Parwani D , et al. An exhaustive perspective on structural insights of SGLT2 inhibitors: a novel class of antidiabetic agent. Eur J Med Chem. 2020;204:112523. 32717480 10.1016/j.ejmech.2020.112523

[3] Wilcox T , De Block C , Schwartzbard AZ , Newman JD . Diabetic agents, from metformin to SGLT2 inhibitors and GLP1 receptor agonists. J Am Coll Cardiol. 2020;75:1956‐1974. 32327107 10.1016/j.jacc.2020.02.056 PMC7219531

[4] electronics Medicine Compendium (eMC) Summary of Product Characteristics (SmPC) . 2020.

[5] Brown E , Rajeev SP , Cuthberston DJ , Wilding JPH . A review of the mechanism of action, metabolic profile and haemodynamic effects of sodium‐glucose co‐transporter‐2 inhibitors. Diabetes Obes Metab. 2019;21:9‐18. 31081592 10.1111/dom.13650

[6] Fioretto P , Zambon A , Rossato M , Busetto L , Vettor R . SGLT2 inhibitors and the diabetic kidney. Diabetes Care. 2016;39:171. 10.2337/dcS15-3006 27440829

[7] Shivakumar O , Sattar N , Wheeler DC . Sodium‐glucose cotransporter 2 inhibitor effects on cardiovascular outcomes in chronic kidney disease. Nephrol Dial Transplant. 2020;35:i43‐i47. 32003831 10.1093/ndt/gfz292 PMC6993195

[8] Sarafidis P , Ortiz A , Ferro CJ , et al. The SGLT‐2 inhibitors for patients with diabetic and non‐diabetic chronic kidney disease: a new era has begun. J Hypertens. 2021;39:1090–1097. 33443971 10.1097/HJH.0000000000002776

[9] Feder D , Gouveia MR , Govato TC , Nassis CD . SGLT2 inhibitors and the mechanisms involved in weight loss. Curr Pharmacol Rep. 2020;6:346‐353.

[10] Giorgino F , Vora J , Fenici P , Solini A . Renoprotection with SGLT2 inhibitors in type 2 diabetes over a spectrum of cardiovascular and renal risk. Cardiovasc Diabetol. 2020;19:196. 33222693 10.1186/s12933-020-01163-9 PMC7680601

[11] Sarafidis P , Ferro CJ , Morales E , et al. SGLT‐2 inhibitors and GLP‐1 receptor agonists for nephroprotection and cardioprotection in patients with diabetes mellitus and chronic kidney disease. A consensus statement by the EURECA‐m and the DIABESITY working groups of the ERA‐EDTA. Nephrol Dial Transplant. 2019;34:208‐230. 30753708 10.1093/ndt/gfy407

[12] Hsia D , Grove O , Cefalu W . An update on sodium‐glucose co‐transporter‐2 inhibitors for the treatment of diabetes mellitus. Curr Opin Endocrinol Diabetes Obes. 2017;24:73‐79. 27898586 10.1097/MED.0000000000000311 PMC6028052

[13] Lahnwong S , Chattipakorn S , Chattipakorn N . Potential mechanisms responsible for cardioprotective effects of sodium–glucose co‐transporter 2 inhibitors. Cardiovasc Diabetol. 2018;17:101. 29991346 10.1186/s12933-018-0745-5 PMC6038192

[14] Bonaventura A , Carbone S , Dixon D , Abbate A , Montecucco F . Pharmacologic strategies to reduce cardiovascular disease in type 2 diabetes mellitus: focus on SGLT‐2 inhibitors and GLP‐1 receptor agonists. J Intern Med. 2019;286:16‐31. 30888088 10.1111/joim.12890

[15] Leon BM , Maddox TM . Diabetes and cardiovascular disease: epidemiology, biological mechanisms, treatment recommendations and future research. World J Diabetes. 2015;6:1246. 26468341 10.4239/wjd.v6.i13.1246 PMC4600176

[16] Zou C‐Y , Liu X‐K , Sang Y‐Q , et al. Effects of SGLT2 inhibitors on cardiovascular outcomes and mortality in type 2 diabetes A meta‐analysis. Medicine. 2019;98:49. 10.1097/MD.0000000000018245 PMC6919451 31804352

[17] Dekkers CCJ , Gansevoort RT . Sodium‐glucose cotransporter 2 inhibitors: extending the indication to non‐diabetic kidney disease? Nephrol Dial Transplant. 2020;35:i33‐i42m. 32003836 10.1093/ndt/gfz264 PMC6993196

[18] Garon SL , Pavlos RK , White KD , Brown NJ , Stone CA Jr , Phillips EJ . Pharmacogenomics of off‐target adverse drug reactions. Br J Clin Pharmacol. 2017;83:1896‐1911. 28345177 10.1111/bcp.13294 PMC5555876

[19] McGill JB , Subramanian S . Safety of sodium‐glucose co‐transporter 2 inhibitors. Am J Med. 2019;132:S49‐S57. 10.1016/j.amjcard.2019.10.029 31741440

[20] Ado Moumouni AN , Robin P , Hillaire‐Buys D , Faillie J‐L . SGLT‐2 inhibitors and ketoacidosis: a disproportionality analysis in the World Health Organization’s adverse drug reactions database. Fundam Clin Pharmacol. 2018;32:216‐226. 29144574 10.1111/fcp.12334

[21] Sato K , Mano T , Iwata A , et al. Subtype‐dependent reporting of stroke with SGLT2 inhibitors: implications from a Japanese Pharmacovigilance Study. J Clin Pharmacol. 2020;60:629‐635. 31792991 10.1002/jcph.1561

[22] Garcia M , Arteche‐Martinez U , Lertxundi U , et al. SGLT2 inhibitors and bladder cancer: analysis of cases reported in the European Pharmacovigilance Database. J Clin Pharmacol. 2021;61:187‐192. 32827151 10.1002/jcph.1722

[23] Interactive Drug Analyses profiles. uk/iDAP/. Accessed October 1, 2020. https://yellowcard.mhra.gov

[24] Cianciolo G , De Pascalis A , Gasperoni L , et al. The off‐target effects, electrolyte and mineral disorders of SGLT2i. Molecules. 2020;25:2757. 32549243 10.3390/molecules25122757 PMC7355461

[25] De Pascalis A , Cianciolo G , Capelli I , Brunori G , La Manna G . SGLT2 inhibitors, sodium and off‐target effects: an overview. J Nephrol. 2021;34(3):673‐680. doi:10.1007/s40620-020-00845-7 32870494

[26] Raschi E , Parisotto M , Foresci E , et al. Adverse events with sodium‐glucose co‐transporter‐2 inhibitors: a global analysis of international spontaneous reporting systems. Nutr Metab Cardiovasc Dis. 2017;27:1098‐1107. 29174026 10.1016/j.numecd.2017.10.008

[27] Raschi E , Poluzzi E , Salvo F , et al. Pharmacovigilance of sodium‐glucose co‐transporter‐2 inhibitors: what a clinician should know on disproportionality analysis of spontaneous reporting systems. Nutr Metab Cardiovasc Dis. 2018;28:533‐542. 29625780 10.1016/j.numecd.2018.02.014

[28] Fuchigami H , Bal MK , Brownson DAC , Banks CE , Jones AM . Voltammetric behaviour of drug molecules as a predictor of metabolic liabilities. Sci Pharm. 2020;88:46.

[29] Ferro CJ , Solkho F , Jalal Z , Al‐Hamid AM , Jones AM . Relevance of physicochemical properties and functional pharmacology data to predict the clinical safety profile of direct oral anticoagulants. Pharmacol Res Perspect. 2020;8:e00603. 32500654 10.1002/prp2.603 PMC7272392

[30] Jalal Z , Cabdi S , Khan N , et al. Sacubitril/Valsartan (Entresto) hospital prescribing in patients with symptomatic chronic HF with reduced ejection fraction: a UK multi‐centre study. J Prescrib Prac. 2019;1:182‐192.

[31] Sandhu D , Antolin AA , Cox AR , Jones AM . Identification of different side effects between PARP inhibitors and their polypharmacological multi‐target rationale. Br J Clin Pharmacol. Epub ahead of print. doi: 10.1111/bcp.15015 34327724

[32] Miljkovic F , Bajorath J . Data‐driven exploration of selectivity and off‐target activities of designated chemical probes. Molecules. 2018;23:2434. 30249057 10.3390/molecules23102434 PMC6222907

[33] Williams DM , Nawaz A , Evans M . Sodium‐glucose co‐transporter 2 (SGLT2) inhibitors: are they all the same? A narrative review of cardiovascular outcome trials. Diabetes Ther. 2021;12:55‐70. 33185854 10.1007/s13300-020-00951-6 PMC7843788

[34] Accessed November 1, 2020. https://www.medicines.org.uk/emc/

[35] ChEMBL Database . Accessed November 1, 2020. https://www.ebi.ac.uk/chembl/

[36] Gaulton A , Hersey A , Nowotka M , et al. The ChEMBL database in 2017. Nucleic Acids Res. 2017;45(D1):D945‐D954. doi:10.1093/nar/gkw1074. PMC: PMC5210557 27899562 PMC5210557

[37] Davies M , Nowotka M , Papadatos G , et al. ChEMBL web services: streamlining access to drug discovery data and utilities. Nucleic Acids Res. 2015;43(W1):W612‐W620. doi:10.1093/nar/gkv352. PMC: PMC4489243 25883136 PMC4489243

[38] Jupp S , Malone J , Bolleman J , et al. The EBI RDF platform: linked open data for the life sciences. Bioinformatics. 2014;30(9):1338‐1339. doi:10.1093/bioinformatics/btt765 24413672 PMC3998127

[39] Hann M . Molecular obesity, potency and other addictions in drug discovery. Med Chem Commun. 2011;2:349.

[40] Freeman‐Cook KD , Hoffman RL , Johnson TW . Lipophilic efficiency: the most important efficiency metric in medicinal chemistry. Future Med Chem. 2013;5:113‐115. 23360135 10.4155/fmc.12.208

[41] Hopkins AL , Keseru GM , Leeson PD , Rees DC , Reynolds CH . The role of ligand efficiency metrics in drug discovery. Nat Rev Drug Discov. 2014;13:105‐121. 24481311 10.1038/nrd4163

[42] Accessed November 5, 2020. https://www.accessdata.fda.gov/drugsatfda_docs/nda/2014/202293Orig1s000PharmR.pdf

[43] Accessed November 5, 2020. https://www.accessdata.fda.gov/drugsatfda_docs/nda/2013/204042Orig1s000PharmR.pdf

[44] Accessed November 5, 2020. https://www.accessdata.fda.gov/drugsatfda_docs/nda/2014/202293Orig1s000PharmR.pdf

[45] Accessed July 29, 2021. https://www.accessdata.fda.gov/drugsatfda_docs/label/2020/209803s002lbl.pdf

[46] Laffel LMB , Tamborlane WV , Yver A , et al. Pharmacokinetic and pharmacodynamic profile of the sodium‐glucose co‐transporter‐2 inhibitor empagliflozin in young people with Type 2 diabetes: a randomized trial. Diabet Med. 2018;35:1096‐1104. 29655290 10.1111/dme.13629 PMC6099360

[47] Alsanosi SMM , Skiffington C , Padmanabhan S . Pharmacokinetic pharmacogenomics. In: Padmanabhan S , ed. Handbook of Pharmacogenomics and Stratified Medicine. Academic Press; 2014:341‐364. ISBN 9780123868831. doi:10.1016/B978-0-12-386882-4.00017-7

[48] Tahara A , Takasu T , Yokono M , Imamura M , Kurosaki E . Characterization and comparison of sodium‐glucose cotransporter 2 inhibitors in pharmacokinetics, pharmacodynamics, and pharmacologic effects. J Pharmacol Sci. 2016;130:159‐169. 26970780 10.1016/j.jphs.2016.02.003

[49] Fediuk DJ , Nucci G , Dawra VK , et al. Overview of the clinical pharmacology of ertugliflozin, a novel sodium‐glucose cotransporter 2 (SGLT2) inhibitor. Clin Pharmacokinet. 2020;59:949‐965. 32337660 10.1007/s40262-020-00875-1 PMC7403171

[50] Devineni D , Polidori D . Pharmacodynamic, and drug‐drug interaction profile of canagliflozin, a sodium‐glucose co‐transporter 2 inhibitor. Clin Pharmacokinet. 2015;54:1027‐1041. 26041408 10.1007/s40262-015-0285-z

[51] Scheen AJ . Pharmacokinetic and pharmacodynamic profile of empagliflozin, a sodium glucose co‐transporter 2 inhibitor. Clin Pharmacokinet. 2014;53:213‐225. 24430725 10.1007/s40262-013-0126-x PMC3927118

[52] Kaischayanula S , Liu X , LaCreta F , Griffen SC , Boulton DW . Clinical pharmacokinetics and pharmacodynamics of dapagliflozin, a selective inhibitor of sodium‐glucose co‐transporter type 2. Clin Pharmacokinet. 2014;53:17‐27. 24105299 10.1007/s40262-013-0104-3

[53] Hawes JJ , Wange R , Guettier J‐M , Godwin E . Non‐Clinical Review(s) – Dapagliflozin. Centre for Drug Evaluation and Research. Accessed November 5, 2020. https://www.accessdata.fda.gov/drugsatfda_docs/nda/2017/209803,209805,209806Orig1s000PharmR.pdf

[54] Alexander SP , Fabbro D , Kelly E , et al. The concise guide to PHARMACOLOGY 2017/18: enzymes. Br J Clin Pharmacol. 2017;174:S272‐S359. 10.1111/bph.13877 PMC5650666 29055034

[55] Alexander SPH , Kelly E , Marrion N , et al. The concise guide to PHARMACOLOGY 2015/16: overview. Br J Clin Pharmacol. 2015;172:5729‐5743. 10.1111/bph.13347 PMC4718217 26650438

[56] Open Prescribing. Accessed October 10, 2020. https://openprescribing.net/

[57] Grabowski B . “*P* < 0.05” might not mean what you think: American Statistical Association clarifies *P* values. J Natl Cancer Inst. 2016;108:djw194. 10.1093/jnci/djw194 PMC5017929 27510514

[58] Caster O , Aoki Y , Gattepaille LM , Grundmark B . Disproportionality analysis for pharmacovigilance signal detection in small databases or subsets: recommendations for limiting false‐positive associations. Drug Saf. 2020;43:479‐487. 32008183 10.1007/s40264-020-00911-w PMC7165139

[59] Tamura Y , Miyagawa H , Yoshida T , Chuman H . Binding interaction of SGLT with sugar and thiosugar by the molecular dynamics simulation. Biochim Biophys Acta. 2015;1848:2799‐2804. 26260238 10.1016/j.bbamem.2015.08.001

[60] Isaji M . SGLT2 inhibitors: molecular design and potential differences in effect. Kidney Int Suppl. 2011;79:S14‐S19. 21358697 10.1038/ki.2010.511

[61] SGLT2 inhibitors: reports of Fournier’s gangrene (necrotising fasciitis of the genitalia or perineum). Accessed November 6, 2020.https://www.gov.uk/drug‐safety‐update/sglt2‐inhibitors‐reports‐of‐fournier‐s‐gangrene‐necrotising‐fasciitis‐of‐the‐genitalia‐or‐perineum

[62] Huang CY , Lee JK . Sodium‐glucose co‐transporter‐2 inhibitors and major adverse limb events: a trial‐level meta‐analysis including 51 713 individuals. Diabetes Obes Metab. 2020;22:2348‐2355. 32744411 10.1111/dom.14159

[63] Hu Y , Bai Z , Tang Y , et al. Fournier gangrene associated with sodium‐glucose cotransporter‐2 inhibitors: a pharmacovigilance study with data from the U.S. FDA Adverse Event Reporting System. J Diabetes Res. 2020;2020:3695101. 32695827 10.1155/2020/3695101 PMC7368210

[64] Paranjape AN , Navale AM . Glucose transporters: physiological and pathological roles. Biophys Rev. 2016;8(1):5‐9. 10.1007/s12551-015-0186-2 PMC5425736 28510148

[65] Song P , Onishi A , Koepsell H , Vallon V . Sodium glucose cotransporter SGLT1 as a therapeutic target in diabetes mellitus. Exp Opin Ther Targets. 2016;20(9):1109‐1125. 10.1517/14728222.2016.1168808 PMC5045806 26998950

[66] Rieg JAD , Rieg T . What does SGLT1 inhibition add: prospects for dual inhibition. Diabetes Obes Metab. 2019;2:43‐52. 10.1111/dom.13630 PMC6516085 31081587

[67] Tazawa S , Yamato T , Fujikura H , et al. SLC5A9/SGLT4, a new Na+‐dependent glucose transporter, is an essential transporter for mannose, 1,5‐anhydro‐D‐glucitol, and fructose. Life Sci. 2005;76:1039‐1050. 15607332 10.1016/j.lfs.2004.10.016

[68] Yamazaki Y , Harada S , Tokuyama S . Sodium‐glucose transport type 3‐mediated neuroprotective effect of acetylcholine suppresses the development of cerebral ischaemic neuronal damage. Neuroscience. 2014;269:134‐142. 24699226 10.1016/j.neuroscience.2014.03.046

[69] Dicembrini I , Tomberli B , Nreu B , et al. Peripheral artery disease and amputations with Sodium‐Glucose Co‐Transporter‐2 (SGLT‐2) inhibitors: a meta‐analysis of randomized controlled trials. Diabetes Res Clin Pract. 2019;153:138‐144. 31150722 10.1016/j.diabres.2019.05.028

[70] Khouri C , Cracowski J , Roustit M . SGLT‐2 inhibitors and the risk of lower‐limb amputation: is this a class effect? Diabetes Obes Metab. 2018;20:1531‐1534. 29430814 10.1111/dom.13255

[71] Tanaka A , Node K . Increased amputation risk with canagliflozin treatment: behind the large cardiovascular benefit? Cardiovasc Diabetol. 2017;16:129. 29025400 10.1186/s12933-017-0611-x PMC5639481

[72] Saliba W , Nitzan O , Chazan B , Elias M . Urinary tract infections in patients with type 2 diabetes mellitus: review of prevalence, diagnosis, and management. Diabetes Metab Syndr Obes. 2015;8:129‐136. 25759592 10.2147/DMSO.S51792 PMC4346284

[73] Fadini GP , Bonora BM , Mayur S , et al. Dipeptidyl peptidase‐4 inhibitors moderate the risk of genitourinary tract infections associated with sodium‐glucose co‐transporter‐2 inhibitors. Diabetes Obes Metab. 2018;20:740‐744. 29053207 10.1111/dom.13130

[74] Ceriello A , Genovese S , Mannucci E , Gronda E . Glucagon and heart in type 2 diabetes: new perspectives. Cardiovasc Diabetol. 2016;15:123. 27568179 10.1186/s12933-016-0440-3 PMC5002329

[75] Capozzi ME , Coch RW , Koech J , et al. The limited role of glucagon for ketogenesis during fasting or in response to SGLT2 inhibition. Diabetes. 2020;69:882‐892. 32005706 10.2337/db19-1216 PMC7171961

[76] SGLT2 inhibitors: monitor ketones in blood during treatment interruption for surgical procedures or acute serious medical illness (published 18 Mar 2020) and in Drug Safety Update volume 13, issue 8: March 2020: 4. [https://www.gov.uk/drug‐safety‐update/sglt2‐inhibitors‐monitor‐ketones‐in‐blood‐during‐treatment‐interruption‐for‐surgical‐procedures‐or‐acute‐serious‐medical‐illnesshttps://assets.publishing.service.gov.uk/government/uploads/system/uploads/attachment_data/file/873524/March‐2020‐PDF.pdf]

[77] Taylor S , Blau J , Rother K . SGLT2 inhibitors may predispose to ketoacidosis. J Clin Endocrinol Metab. 2015;100:2849‐2852. 26086329 10.1210/jc.2015-1884 PMC4525004

[78] Pedersen MG , Ahlstedt I , Hachmane MFEL , Göpel SO . Dapagliflozin stimulates glucagon secretion at high glucose: experiments and mathematical simulations of human A‐cells. Sci Rep. 2016;6:31214. 27535321 10.1038/srep31214 PMC4989223

[79] Suga T , Kikuchi O , Kobayashi M , et al. SGLT1 in pancreatic α cells regulates glucagon secretion in mice, possibly explaining the distinct effects of SGLT2 inhibitors on plasma glucagon levels. Mol Metab. 2019;19:1‐12. 30416006 10.1016/j.molmet.2018.10.009 PMC6323192

[80] Vuppalanchi R . Metabolism of drugs and xenobotics. In: Saxena R , ed. Prac Hep Path; A Diag Appr: A Diag Appr. Philadelphia, PA: Elsevier; 2011:319‐326. 10.1016/B978-0-323-42873-6.00022-6

[81] Pattanawonga A , Chau N , Rowland A , Miners J . The inhibition of human UDP‐glucuronosyltransferase (UGT) enzymes by canagliflozin and dapagliflozin: implications of drug‐drug interactions. Drug Metab Dispos. 2015;43:1468‐1476. 26180128 10.1124/dmd.115.065870

[82] Shitara Y . Clinical importance of OATP1B1 and OATP1B3 in drug‐drug interactions. Drug Metab Pharmacokinet. 2011;26:220‐227. 21297316 10.2133/dmpk.DMPK-10-RV-094

[83] Hazell L , Shakir SAW . Under‐reporting of adverse drug reactions a systematic review. Drug Saf. 2006;29:385‐396. 16689555 10.2165/00002018-200629050-00003

[84] Pariente A , Gregoire F , Fourrier‐Reglat A , Haramburu F , Moore N . Impact of safety alerts on measures of disproportionality in spontaneous reporting databases. The Notoriety Bias. Drug Saf. 2007;30:891‐898. 17867726 10.2165/00002018-200730100-00007

[85] Arora A , Jalali RK , Vohora D . Relevance of the Weber effect in contemporary pharmacovigilance of oncology drugs. Ther Clin Risk Manag. 2017;13:1195‐1203. 28979130 10.2147/TCRM.S137144 PMC5602442

[86] Mestres J , Gregori‐Puigjané E , Valverde S , Solé RV . Data completeness–the Achilles heel of drug‐target networks. Nat Biotechnol. 2008;26:983‐984. 18779805 10.1038/nbt0908-983

[87] Antolin AA , Tym JE , Komianou A , Collins I , Workman P , Al‐Lazikani B . Objective, quantitative, data‐driven assessment of chemical probes. Cell Chem Biol. 2018;25:194‐205.e5. 29249694 10.1016/j.chembiol.2017.11.004 PMC5814752

