# Supplementary material for: Polypharmacology of clinical sodium glucose co‐transport protein 2 inhibitors and relationship to suspected adverse drug reactions
Source: Pharmacol Res Perspect. 2021 Sep 29;9(5):e00867. doi: 10.1002/prp2.867 (PMC8480305; doi:10.1002/prp2.867)
Supplement: Supplementary file 1 — Supplementary Material [file PRP2-9-e00867-s001.docx]

**SUPPORTING INFORMATION**

**Polypharmacology of clinical sodium glucose co-transport protein 2 inhibitors and relationship to suspected adverse drug reactions**

Karan Matharu,^1^ Kiran Chana,^1^ Charles J. Ferro,^2^ Alan M. Jones^1*^

^1^ School of Pharmacy, Institute of Clinical Sciences, College of Medical and Dental Sciences, University of Birmingham, Edgbaston, Birmingham, B15 2TT, United Kingdom

^2^ Birmingham Cardio-Renal Group, Institute of Cardiovascular Sciences, College of Medical and Dental Sciences, University of Birmingham B15 2TT, United Kingdom

**Correspondence**

Dr A. M. Jones, School of Pharmacy, Institute of Clinical Sciences, College of Medical and Dental Sciences, University of Birmingham, Birmingham, B15 2TT, United Kingdom. Email: [a.m.jones.2@bham.ac.uk](mailto:a.m.jones.2@bham.ac.uk)

Kingdom

**CONTENTS**

Table S1 page 2

Table S2 page 5

Chart S1 page 6

Chart S2 page 7

**Table S1.** Statistical Summary for all SGLT-2 inhibitors’ ADRs (*χ^2^* test). (-) a *p* value was not determined.

| **ADRs** | **All SGLT-2 inhibitors** | **Empagliflozin vs Dapagliflozin** | **Empagliflozin vs Canagliflozin** | **Dapagliflozin vs Canagliflozin** |
| --- | --- | --- | --- | --- |
| All ADRs | <0.05 | <0.05 | <0.05 | 0.752 |
| **Gastrointestinal System** |  |  |  |  |
| Total ADRs | 0.331 | 0.230 | 0.146 | 0.793 |
| Nausea and Vomiting | 0.723 | 0.592 | 0.420 | 0.985 |
| **General System Disorders** |  |  |  |  |
| Total ADRs | 0.200 | 0.100 | 0.096 | 0.882 |
| Asthenic conditions | 0.701 | 0.411 | 0.497 | 0.264 |
| **Hepatobiliary Disorders** |  |  |  |  |
| Total ADRs | 0.827 | 0.547 | 0.767 | 0.745 |
| **Infections and Infestations** |  |  |  |  |
| Total ADRs | 0.052 | 0.170 | <0.05 (0.015) | 0.264 |
| Fungal Infections | 0.058 | 0.378 | 0.309 | 0.883 |
| Urinary Tract Infections | 0.118 | 0.355 | 0.052 | 0.248 |
| Fournier’s gangrene | 0.946 | 0.751 | 0.920 | 0.827 |
| **Renal System** |  |  |  |  |
| Total ADRs | 0.274 | 0.113 | 0.185 | 0.782 |
| Urinary Disorders | 0.453 | 0.241 | 0.251 | 0.979 |
| Acute Kidney Injury (AKI) | 0.841 | 0.622 | 0.570 | 0.938 |
| **Reproductive System** |  |  |  |  |
| Total ADRs | 0.593 | 0.342 | 0.767 | 0.504 |
| Penile Disorders | 0.989 | 0.930 | 0.884 | 0.953 |
| Balanoposthitis | 0.983 | 0.859 | 0.949 | 0.909 |
| Vulvovaginal Disorders | 0.804 | 0.504 | 0.627 | 0.842 |
| **Skin and Subcutaneous** |  |  |  |  |
| Total ADRs | 0.289 | 0.573 | 0.119 | 0.425 |
| Skin disorders | 0.292 | 0.474 | 0.126 | 0.396 |
| Angioedema | 0.988 | 0.954 | 0.925 | 0.881 |
| **Surgical Procedures** |  |  |  |  |
| Total ADRs | 0.548 | 0.907 | 0.382 | 0.400 |
| Foot Amputations | 0.368 | - | 0.317 | 0.317 |
| Limb Amputations | 0.368 | 0.564 | 0.157 | 0.317 |
| Toe Amputations | 0.477 | 0.989 | 0.360 | 0.355 |
| Leg Amputations | 0.913 | 0.809 | 0.852 | 0.708 |
| **Nervous System** |  |  |  |  |
| Total ADRs | 0.377 | 0.208 | 0.191 | 0.961 |
| Neurological disorders | 0.525 | 0.317 | 0.280 | 0.933 |
| **Metabolic Disorders** |  |  |  |  |
| Total ADRs | 0.307 | 0.197 | 0.214 | 0.961 |
| DKA | 0.444 | 0.202 | 0.477 | 0.567 |
| EuDKA | 0.693 | 0.995 | 0.484 | 0.488 |
| **Muscoskeltal System** |  |  |  |  |
| Total ADRs | 0.388 | 0.254 | 0.170 | 0.802 |
| Back Pain | 0.739 | 0.561 | 0.943 | 0.520 |

**Table S2.** Data for the pharmacological activity of all SGLT-2 inhibitors.( - ) Represents no data was reported. * these targets are mentioned in the literature but no IC_50_ is reported.

| **On- and Off-Target Activities** | **Empagliflozin** | **Dapagliflozin** | **Canagliflozin** | **Ertugliflozin** |
| --- | --- | --- | --- | --- |
| **SGLT (SLC5A Gene) IC_50_ (nM)** |  |  |  |  |
| SGLT-2 | 3.1 | 1.2 | 2.7 | 0.87 |
| SGLT-1 | 8300 | 1400 | 710 | 1960 |
| Selectivity (SGLT2:SGLT1) | ≅2500 fold | ≅1200 fold | ≅260 fold | ≅2200 |
| SGLT-4 | 11000 | 9100 | 7900 | - |
| SGLT-5 | 1100 | 820 | 1700 | - |
| SGLT-6 | 2000 | 1300 | 240 | - |
| ***CYP450 Isoforms -* IC_50_ (nM)** |  |  |  |  |
| 1A2 | >100000 | >40000 | - | - |
| 2B6 | >50000 | >40000 | - | - |
| 2C8 | >100000 | >40000 | 37500 | >30000 |
| 2C9 | ≅150000 | >40000 | 27500 | - |
| 2C19 | >150000 | >40000 | 19500 | - |
| 2D6 | >150000 | >40000 | 1320 | - |
| 3A4 | >150000 | >40000 | - | >30000 |
| 3A5 | - | - | 27000 | >30000 |
| **SLC22 - subfamily IC_50_ (nM)** |  |  |  |  |
| OATP1BI | 71800 | 69300 | - | 35400 |
| OATP1B3 | 58600 | 8000 | - | 150700 |
| OATP2B1 | 45200 | - | - | - |
| OAT3 | 295000 | 33000 | - | 70000 |
| OCT1a | - | - | - | 53000 |
| OCT2a | >1000000 | - | - | 917000 |
| **UDP - *UGT* Isoforms IC_50_ (nM)** |  |  |  |  |
| UGT1A1 | >50000 | >50000 | - | >39000 |
| UGT1A9 | - | 39000-66000 | - | - |
| **hERG IC_50_ (nM)** | >30000 | >30000 | - | 59000 |
| GLUT1 | - | >20000 | >1000 | - |
| GLUT4, AMPK, SERT1, NHE3, NHE1* | - | - | - | - |

**Chart S1.** Number of reported ADRs per organ class. Key: empagliflozin (blue), dapagliflozin (orange), and canagliflozin (red).

**Chart S2.** Number of reported fatalities per organ class. Key: empagliflozin (blue), dapagliflozin (orange), and canagliflozin (red).
